# Methicillin-Resistant Staphylococcus aureus (MRSA)-Positive Pericardial Abscess Presenting in a Hemodialysis Patient

**DOI:** 10.7759/cureus.10411

**Published:** 2020-09-12

**Authors:** Christopher C Zarour, Mario Dervishi, Daniel Fuguet, Zahraa Al-bahbahanee

**Affiliations:** 1 Radiology, St. Joseph Mercy Hospital, Pontiac, USA; 2 Radiology, American University of the Caribbean School of Medicine, Cupecoy, SXM; 3 Internal Medicine, University of Baghdad Al-Kindy College of Medicine, Baghdad, IRQ

**Keywords:** pericardial abscess, pericardial cyst, methicillin-resistant staphylococcus aureus, end-stage renal disease.

## Abstract

Purulent pericarditis is an uncommon infection of the pericardial space that can very rarely present as a pericardial abscess. Infection by hematogenous spread in dialysis patients is among the predisposing risk factors that can lead to purulent pericarditis. Herein, we present a pericardial abscess case due to methicillin-resistant *Staphylococcus aureus* (MRSA) infection in a male patient with end-stage renal disease (ESRD) on hemodialysis. The patient has a past surgical history of two prior pericardial effusions due to uremic pericarditis.

## Introduction

Purulent pericarditis is an extremely rare infectious etiology affecting the pericardial space characterized by gross pus in the pericardium. It is mostly associated with nosocomial hematogenous spread, as can be seen in dialysis or surgical interventions such as pericardial windows [1]. 

Predisposing risk factors of purulent pericarditis include pericardial effusion (infectious or inflammatory), chronic kidney disease, immunocompromised status, alcohol abuse, cardiothoracic surgery, and trauma [1]. It has been revealed that patients with purulent pericarditis also had preexisting pericardial diseases as a result of uremia, malignancy, or collagen vascular diseases [1,2].

Purulent pericarditis as an acute illness is characterized by high fever, tachycardia, cough, and chest pain. However, rarely no signs of sepsis of may be appreciated, which makes the diagnosis extremely difficult [3]. Infection usually involves the entire pericardium, but can rarely present as a pericardial abscess [4]. 

Two-dimensional echocardiography and CT are crucial in early detection to decrease mortality [4]. However, there are many mimics that may resemble pericardial abscess, and as a result imaging cannot be used alone. Diagnosis must be established by image-guided needle aspiration of the fluid collection in the pericardium, with appropriate fluid cytology and culture, to ensure the offending etiology is pinpointed and immediate therapy is initiated. 

## Case presentation

A 42-year-old male with a past medical history of end-stage renal disease (ESRD) on hemodialysis presented to the emergency department (ED) with severe anemia and hypotension after hemodialysis. 

**Figure 1:**
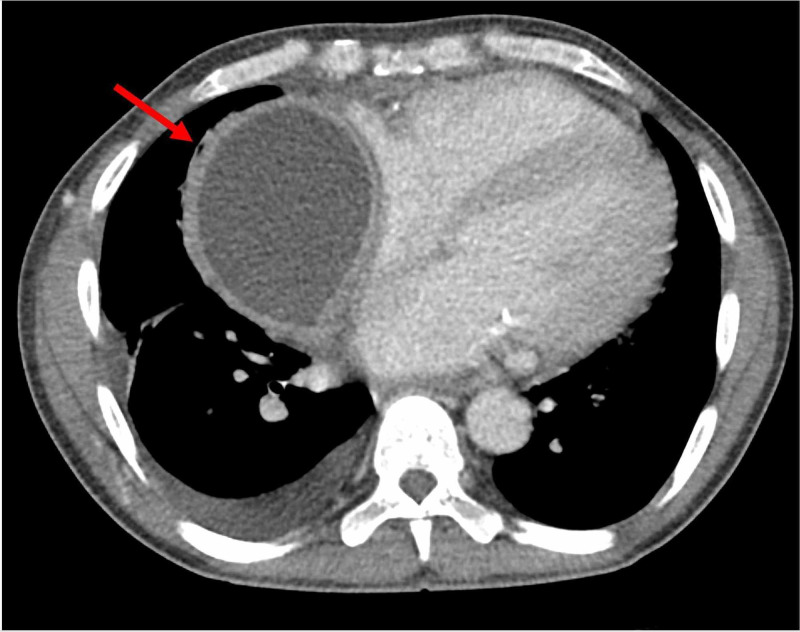
Pericardial Abscess: Axial CT Chest With IV Contrast.

**Figure 2:**
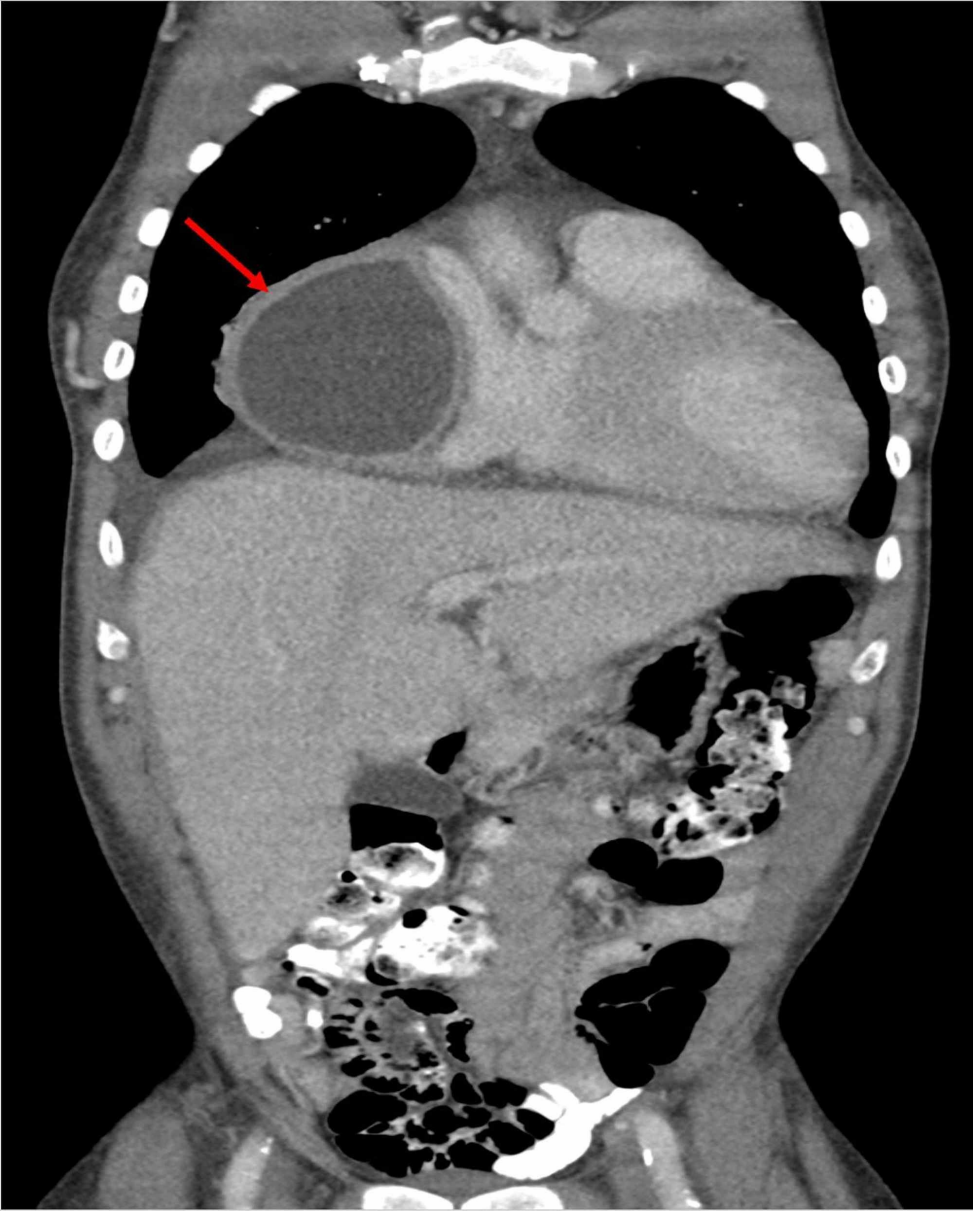
Pericardial Abscess: Coronal CT Chest, Abdomen, and Pelvis With IV and Oral Contrast.

**Figure 3:**
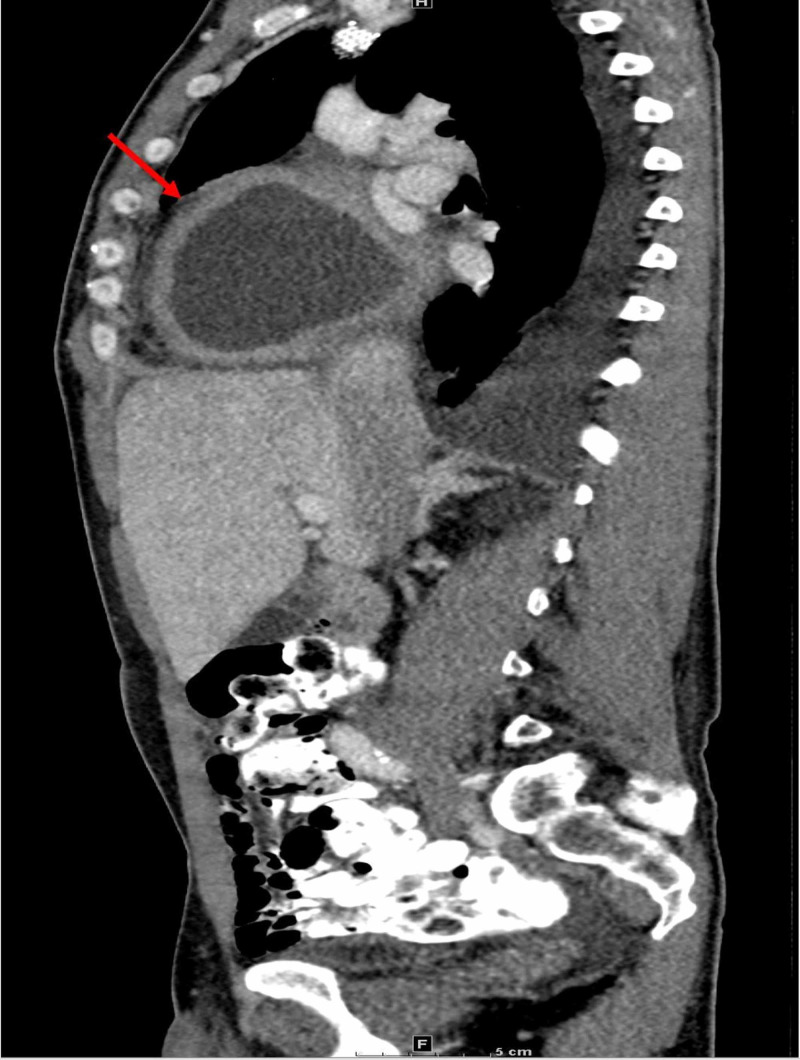
Pericardial Abscess: Sagittal CT Chest, Abdomen, and Pelvis With IV and Oral Contrast.

On physical examination, the patient appeared in no acute distress, while denying fever, chills, nausea, vomiting, or excessive weight loss. His temperature was elevated at 101.2 degrees Fahrenheit. Lab values were unremarkable. With a past history of two pericardial windows attributed to uremic pericarditis/pericardial effusion, a CT of the chest, abdomen and pelvis with intravenous and oral contrast was performed (Figures 1-3). The CT demonstrated a thick-walled right cardiophrenic angle cystic mass measuring 9 x 11 x 11 cm (anteroposterior x transverse x craniocaudal dimensions) causing mass effect on the right atrium and ventricle. An emergent interventional radiology consultation was placed, and a CT-guided needle aspiration was performed. Fluid analysis and culture yielded positive methicillin-resistant *Staphylococcus aureus*, in keeping with a pericardial abscess. The pericardial abscess was drained and treatment with vancomycin ensured a complete recovery. 

## Discussion

Pericardial abscess has life-threatening consequences due to the risk of bacteremia and mass effect on the adjacent heart structures, resulting in severe hypotension [5]. The importance of recognizing such infections is dire; however, it can be challenging, as signs and symptoms of sepsis may seize to exist [5,6]. Therefore, radiology imaging and intervention becomes a crucial component for an accurate diagnosis to ensure immediate treatment. Pericardial masses are often initially detected with echocardiography, but CT and MRI can be warranted for further evaluation or characterization to aid in an accurate diagnosis of various pericardial masses [7].

Focal pericardial effusions, ventricular aneurysms, pericardial cysts, and fibromas all present mass effect on the heart contour, which is appreciated on radiological imaging [4]. The challenge then becomes differentiating a cyst from an infectious etiology, like an abscess, because cystic fluid and an abscess are similar in attenuation. In such a scenario, aspiration of the fluid with appropriate cytology and culture is required to differentiate the two [8,9].

Pericardial mass is a rare etiology, and becomes even rarer with a superimposed *Staphylococcus aureus* infection, with very few other cases documented [4]. Our patient’s history of ESRD managed with hemodialysis and two previous pericardial effusions secondary to uremic pericarditis are all risk factors that can lead to purulent pericarditis.

## Conclusions

A pericardial abscess is a rare etiology affecting the heart and is considered a life-threatening complication. Prompt diagnosis with appropriate imaging, CT of the chest with intravenous contrast, is required to fully assess the nature of the disease process and to identify any aliment that may impact the patients stability. Definitive diagnosis is required by image-guided technique with fluid analysis. This allows for immediate implementation of therapy to ensure patient care.
